# Low‐head dams induce biotic homogenization/differentiation of fish assemblages in subtropical streams

**DOI:** 10.1002/ece3.9156

**Published:** 2022-07-30

**Authors:** Qiang Li, Yuzhou Zhang, Ruolan Wang, Ling Chu, Yuru Li, Yunzhi Yan

**Affiliations:** ^1^ Collaborative Innovation Center of Recovery and Reconstruction of Degraded Ecosystem in Wanjiang Basin Co‐founded by Anhui Province and Ministry of Education, and School of Ecology and Environment Anhui Normal University Wuhu China; ^2^ College of Fisheries Ocean University of China Qingdao China

**Keywords:** biotic homogenization and differentiation, low‐head dam, stream fish, taxonomic and functional diversities

## Abstract

Extensive distribution of widespread species and the loss of native species driven by anthropogenic disturbances modify community similarity, resulting in a decrease or increase in community distinctiveness. Data from four basins in the Wannan Mountains, China, were used to evaluate the effects of low‐head dams on patterns of fish faunal homogenization and differentiation based on abundance data. We aimed to examine the spatial changes in taxonomic and functional similarities of fish assemblages driven by low‐head dams and to examine whether the changes in the similarity of fish assemblages differed between taxonomic and functional components. We found that low‐head dams significantly decreased the mean taxonomic similarity but increased the mean functional similarity of fish assemblages in impoundments using abundance‐based approaches, suggesting that taxonomic differentiation accompanied functional homogenization in stream fish assemblages. These results show the importance of population abundance in structuring fish faunal homogenization and differentiation at small scales, especially when the major differences among assemblages are in species abundance ranks rather than species identities. Additionally, we also found only a weak positive correlation between changes in mean taxonomic and functional similarities, and partial pairs exhibited considerable variation in patterns of fish faunal homogenization and differentiation for taxonomic and functional components. In conclusion, this study highlighted that the observed taxonomic differentiation of current fish assemblages (short‐term phenomenon) is probably an early warning sign of further homogenization in regions where native species are completely predominated and that changes in taxonomic similarity cannot be used to predict changes in functional similarity.

## Graphical abstract

In this study,We aimed to (1) quantify the spatial changes in taxonomic and functional similarities of fish assemblages driven by low‐head dams, and (2) examine whether the direct changes in taxonomic and functional similarities of fish assemblages differ in their responses to low‐head dams.

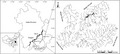

## 1 INTRODUCTION

Anthropogenic disturbances accelerate the replacement rates of native species with widespread species, resulting in a dramatic reconfiguration of current biodiversity (Toussaint et al., 2014; Villéger et al., 2014; Zhang et al., 2019). These disturbances alter not only the number of species in each locality (α‐diversity) (Sax & Gaines, 2003) but also the species composition among localities (β‐diversity) (Erős et al., 2020; Olden et al., 2018; Villéger et al., 2011). Consequently, species losses and gains can increase the community similarity over time (biotic homogenization; Olden & Rooney, 2006). Conversely, biotic differentiation indicates a decrease in community similarity over time (Olden & Poff, 2004). Changes will continue to occur as new species become established and others are lost, causing the formation of “novel communities” (Hobbs et al., 2006), which have notable ecological and evolutionary implications (Olden et al., 2004).

Taxonomic homogenization and differentiation are typically quantified by treating all species identically, despite the fact that they may have different ecological functions (Olden et al., 2018; Rahel, 2000; Taylor, 2010). Functional diversity, as an important component of biodiversity, represents the variation in the functional traits of all species within a community that influences the composition and ecosystem function (Tilman, 2001). Given the functional complementarity and/or redundancy among species traits, functional diversity may not necessarily coincide with patterns of taxonomic diversity under anthropogenic disturbances (Campbell & Mandrak, 2020; Pool & Olden, 2012; Villéger et al., 2014). For instance, Villéger et al. (2014) found that functional homogenization of freshwater fishes in Europe exceeded taxonomic homogenization six times, and approximately 40% of the paired communities showed taxonomic differentiation but functional homogenization. Furthermore, a pair of assemblages that exhibit taxonomic differentiation (an increase in dissimilarity) can actually be functionally homogenized when widespread species have similar functional roles, such as ecological redundancy of traits, when they are introduced into new geographical locations (Pool & Olden, 2012). In contrast, taxonomic homogenization may be accompanied by functional differentiation owing to the loss of unique species with similar functional roles in each community (Campbell & Mandrak, 2020). Therefore, the analysis of changes in functional dissimilarity is complementary to the assessment of changes in taxonomic dissimilarity (Villéger et al., 2014). Assessing the combination of taxonomic and functional components will permit a better understanding of the potential consequences of biodiversity changes on ecosystem functioning under pressure.

Freshwater fish are among the most intensively threatened by anthropogenic disturbances such as dam construction, habitat alteration, and habitat fragmentation (Couto et al., 2021; Dias et al., 2017; Reid et al., 2019). Dams obstruct the dispersal and migration of organisms and modify habitat conditions, both of which are directly linked to patterns of speciation and invasion (Johnson et al., 2008; Petesse & Petrere, 2012; Smith et al., 2017). In addition, dams can break down geographic constraints from physical barriers by allowing the dispersal of fish into systems outside their natural range (Clavero et al., 2013; Vitule et al., 2012) and may contribute to biotic homogenization through habitat changes and homogenization (Daga et al., 2015; Rahel, 2007; Vitule et al., 2012). Low‐head dams have a hydraulic height of <15 m and typically are overflow or spillway structures (Brewitt & Colwyn, 2020; Poff & Hart, 2002), which are ubiquitous human disturbances in headwater streams (Jumani et al., 2020). Similar to large dams, low‐head dams fragment river ecosystems, constrain the upstream movement of fish, create impoundments in place of running water, and alter local habitats (water deepening, flow slowing, and substrate size decrease) (Fencl et al., 2015; Hitchman et al., 2018; Yan et al., 2013). Until now, whether low‐head dams can promote biotic homogenization and differentiation with the same ecological effect as that of larger dams has received less attention (Bu et al., 2017; Liu et al., 2019).

Previous studies on fish homogenization and differentiation driven by low‐head dams have primarily focused on changes in the taxonomic similarity of assemblages, but two important knowledge gaps remain. First, whether the taxonomic and functional components of fish assemblage similarities differ in their responses to low‐head dams remains unclear. Accounting only for the taxonomic component may mask whether a community is functionally saturated when communities have the same number of species but occupy different portions of the functional space (Villéger et al., 2014). Many circumstances, such as geographic barriers and harsh environmental conditions, may obstruct the functional saturation of a community (Głowacki & Penczak, 2013; Mateo et al., 2017). Second, limited empirical evidence has shown the varying role of population abundance in structuring homogenization and differentiation of fish assemblages (Cassey et al., 2008; Legendre, 2014). Only some studies have assessed the role of species abundance in defining the trends towards homogenization and differentiation due to limitations in sampling and accessibility of abundance data (Cassey et al., 2008). Although incidence‐based approaches may play a crucial role when communities differ mostly in species composition and are geographically far apart on global or regional scales (Legendre, 2014), abundance‐based approaches may exhibit higher sensitivity in structuring homogeneous or heterogeneous patterns when species composition differs primarily in terms of species abundance in small spatial extents (Cassey et al., 2008; Dai et al., 2020; Liu et al., 2019). The population size (abundance) of the same species may differ substantially across locations, and the number of individuals is not equally distributed across different species in the same locations. Particularly at small scales, incidence‐based approaches may underestimate the functions of dominant species in biotic homogenization if species abundance data are overlooked (Dai et al., 2020; Liu et al., 2019).

In the present study, we examined the effects of low‐head dams on patterns of fish faunal homogenization and differentiation in headwater streams based on abundance data collected from 53 impoundments created by low‐head dams (treatment sites) and 53 free‐flowing segments below low‐head dams (reference sites) within first‐order streams (stream order based on Strahler, 1957) in the Wannan Mountains, China. We aimed to (1) quantify the spatial changes in taxonomic and functional similarities of fish assemblages driven by low‐head dams, and (2) examine whether the direct changes in taxonomic and functional similarities of fish assemblages differ in their responses to low‐head dams.

## 2 MATERIALS AND METHODS

### 2.1 Study area

The Wannan Mountains, located in the south of Anhui Province, China, are mainly composed of Jiuhua, Huangshan, and Tianmu Mountains, with the highest elevations of 1342 m, 1841 m, and 1787 m, respectively. Owing to the influence of the subtropical monsoon climate, this area is characterized by asymmetric seasonal temperature and precipitation distributions. The annual air temperature ranges from −2.1°C (January) to 27.5°C (July) with a mean of 17.8°C. Annual rainfall is 1100–2500 mm/year, with a mean of approximately 1900 mm/year, of which more than 60% occurs from May to August. The upland headwater streams in this area are highly complex and abundant, and are confluent to the Xin'an, Chang, Qiupu, and Qingyi Rivers, among which the Xin'an River belongs to the Qiantang watershed, and the other three rivers belong to the Yangtze River.

**FIGURE 1:**
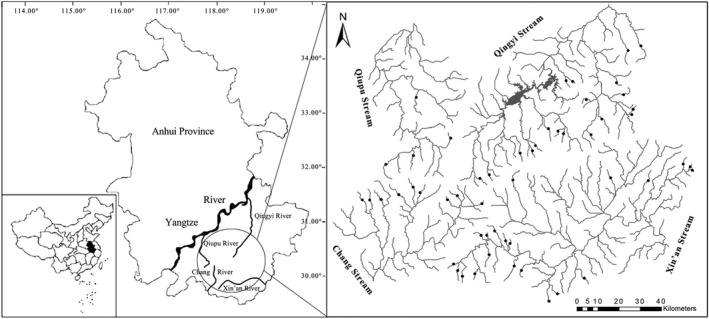
Sampling locations of the four basins of Wannan Mountains, China. Black spots represent the spatial positions of the low‐head dams surveyed in this study. Two sampling sites were set at each dam, including the impoundment area and downstream free‐flowing segment.

Fifty‐three low‐head dams in the first‐order headwater streams of the Wannan Mountains were surveyed, of which 21, 9, 4, and 19 low‐head dams were located in the Xin'an, Chang, Qiupu, and Qingyi Rivers, respectively (Figure 1). Overall, low‐head dams (<5 m in height) were built in the tributaries for agricultural irrigation and water supply in this region (Li et al., 2021; Yan et al., 2013). Given the constraints of time and lack of historical data, biotic homogenization can be studied effectively as a spatial process (Dar & Reshi, 2014). Spatial patterns may also provide tentative evidence for changes in current fish assemblages (Liu et al., 2019). As fish dispersal is restricted to via‐watercourse routes in river ecosystems, low‐head dams can affect stream fish assemblages by changing habitat conditions and blocking upstream movement (Porto et al., 1999; Yan et al., 2013). Compared with the free‐flowing segments below dams, choosing the references above dams usually ignores dams that block fish movement, which may underestimate the ecological effects of low‐head dams on current fish assemblages (Li et al., 2021). Therefore, two types of sampling sites were set for each low‐head dam: impoundments created by low‐head dams (treatment sites) and free‐flowing segments below the low‐head dams (reference sites). In addition, the effects of low‐head dams on geomorphological footprints are often less than 2 km (Fencl et al., 2015), and free‐flowing segments are ensured at least 2 km from the dams.

### 2.2 Field survey

A total of 106 sites (53 impoundments and 53 free‐flowing segments) were sampled across the Wannan Mountains during the wet season (August 2015). The highland stream discharges in this region are characterized by flashy pulses with high‐flow conditions during the wet season (Yan et al., 2013). Following standardized methods such as stream length and sampling time described by Li et al. (2021), stream fish assemblages were sampled at each site using a backpack electrofishing unit (CWB‐2000 P, China; 12 V input and 250 V output) by wading in two passes. Based on Nelson's (2006) classification method, all fishes were identified in the field and counted. At least 20 adult specimens of each species were used to measure the traits, and all adult specimens were measured when the species contained fewer than 20 adult individuals. Whenever possible, only a fraction of the dominant species were returned to the sampling sites if alive, and the remaining species were retained for further laboratory identification and measurement. Fish handling procedures were performed according to the bioethical protocols established by the Anhui Normal University Animal Ethics Committee.

We characterized the local habitat conditions at each sampling site using seven habitat variables: wetted width (m), water depth (m), current velocity (m/s), dissolved oxygen (mg/L), water temperature (°C), substrate coarseness, and heterogeneity. Wetting width was measured along three equally spaced transects across the surveyed stream channel. Water depth was measured at three equally spaced points along each transect. Current velocity was quantified at 60% of the water depth at each point using an FP111 (USA). Dissolved oxygen and water temperatures were measured using a YSI Professional Plus meter (USA). The substrate was quantified within each sample site and divided into 10 cross sections based on the size‐class frequency method of Bain et al. (1985): particle size 0 = 0–0.059 mm, 1 = 0.06–1 mm, 2 = 2–15 mm, 3 = 16–63 mm, 4 = 64–256 mm, and 5 = >256 mm. Mean and standard deviation of substrate values were used as the indices of substrate coarseness and heterogeneity, respectively.

### 2.3 Measurement of species trait

Choosing appropriate functional traits can provide valuable insights into the relationships between environmental changes, fish functional diversity, and ecosystems (Troia & McManamay, 2020; Villéger et al., 2017). As habitat alteration and fragmentation associated with low‐head dams can potentially affect habitat guilds and trophic structures of fish assemblages in headwater streams (Fencl et al., 2017; Helms et al., 2011; Smith et al., 2017), we estimated the functional diversity of each community by measuring the most commonly available traits of fish species related to feeding, locomotion, and habitat preferences (Albouy et al., 2011). We measured a series of fish traits in the laboratory and used them to calculate seven morphological ratio traits: (1) eye size; (2) oral gape position; (3) oral gape shape; (4) gut length; (5) caudal peduncle throttling; (6) body transversal shape; and (7) body depth (Appendix S1). At least 20 adult specimens of each species were used to measure these traits, and all adult specimens were measured when the species contained <20 adult individuals.

### 2.4 Statistical analyses

According to Scott and Helfman (2001) and Liu et al. (2019), we identified native and native‐invasive fish for each fish species collected in this study. In particular, native species with high degrees of endemism are typically adapted to cool, clear, and lotic conditions in mountain streams, whereas native‐invasive species occurring mainly in large streams are likely to be potential invaders of degraded headwater streams, which are highly adapted to warm and nutrient‐ and sediment‐rich conditions (Dala‐Corte et al., 2019; Liu et al., 2019; Scott & Helfman, 2001). From fish data, the frequency of occurrence (*FO*) and relative abundance (*RA*) was calculated for each species as *FO*_i_ = 100(*S*_i_/*S*)% and *RA*_i_ = 100(*N*_i_/*N*)%, where *S*_i_ and *S* are the numbers of samples for which species *i* was collected, and of the total samples, *N*_i_ and *N* are the individual numbers of species *i* and total fish species, respectively. Based on the number of species and number of samples (reference and treatment sites), species accumulation curves were constructed using the “*vegan*” package in R software (R Core Team, 2018; Figure S1).

**FIGURE 2:**
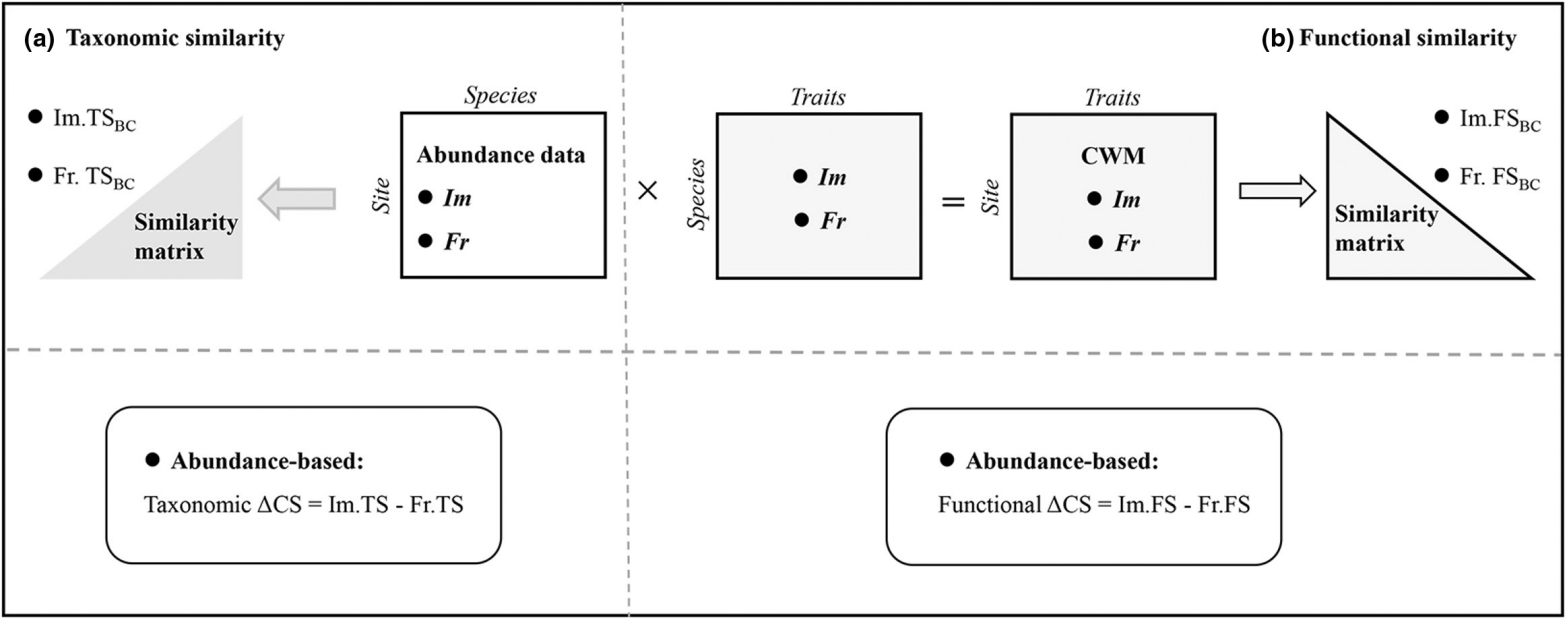
Framework summarizing datasets and statistical analyses for (a) taxonomic similarity and (b) functional similarity. (a) Taxonomic similarity: The *site* × *species* matrices were created separately for each habitat type, i.e., impoundments (*Im*) and free‐flowing segments (*Fr*), and converted into taxonomic similarity matrices (*TS*) based on abundance data. Changes in pairwise taxonomic similarities (taxonomic ΔCS) were measured as impoundment similarity (*Im.TS*) minus the similarity of free‐flowing segments (*Fr.TS*). (b) Functional similarity: The *site×traits* matrices (CWM) for abundance‐based data were created for each habitat type, impoundments (*Im*), and free‐flowing segments (*Fr*), and converted into functional similarity matrices (*FS*) for the impoundments (*Im.FS*) and free‐flowing segments (*Fr.FS*). Changes in pairwise functional similarities (functional ΔCS) were measured as impoundments similarity (*Im.FS*) minus the similarity of free‐flowing segments (*Fr.FS*) (adapted from Baiser & Lockwood, 2011; Pool & Olden, 2012; Daga et al., 2020).

Taxonomic similarity of fish assemblages was quantified using the Bray–Curtis similarity index. Specifically, *site*× *species* matrices were created separately for both the impoundments (*Im*) and free‐flowing segments (*Fr*) based on abundance data (Figure 2a). Then, the taxonomic similarity was calculated separately for each habitat (impoundments: *Im.TS*; free‐flowing segments: *Fr.TS*). Changes in pairwise taxonomic similarity (taxonomic ΔCS) were calculated between the impoundments and free‐flowing segments as follows: taxonomic ΔCS = *Im.TS* − *Fr.TS* (Figure 2a). For functional similarity, we first obtained the community‐weighted mean of trait values per site (CWM) for both impoundments and free‐flowing segments using abundance data. *Site* × *trait* matrices (CWM) were created by multiplying the *site* × *species* matrix and *species×trait* matrix for each habitat type (Figure 2b). The functional similarity was separately calculated for the impoundment assemblages (*Im.FS*) and assemblages sampled in the free‐flowing segments (*Fr.FS*) according to the Euclidean distance (Daga et al., 2020). Following the same approach used for taxonomic analysis, changes in pairwise functional similarity (functional ΔCS) were calculated as follows: functional ΔCS = *Im.FS* − *Fr.FS* (Figure 2b). Positive ΔCS values indicate taxonomic or functional homogenization, whereas negative values indicate differentiation (Baiser & Lockwood, 2011; Daga et al., 2020; Pool & Olden, 2012). All calculations were performed using the “*FD*” package (Laliberté et al., 2014) and “*vegan*” package in R software (Oksanen et al., 2013).

Both principal component analysis (PCA) and permutational multivariate analysis of variance (PERMANOVA) were used to test the differences in local habitat between the impoundments and free‐flowing segments based on standardized environmental variables. We further used the permutational analysis of multivariate dispersions (PERMDISP; Anderson, 2006) to test the differences in environmental heterogeneity between the two habitat types. Using analysis of variance (ANOVA) F‐statistics, PERMDISP compares among‐group differences in the distance from observations to their group centroid, and the significance was tested using permutations of least‐squares residuals (Heino et al., 2013). Using log_10_ (*x* + 1) transformed fish abundance data, a one‐way analysis of similarity (ANOSIM) was used to identify the discrete spatial variation in fish assemblages between the two habitat types. The contribution of each species to the differences among assemblage groups was assessed using similarity percentages (SIMPER). The above analyses were performed using the “*vegan*” package of R software (R Core Team, 2018) (Oksanen et al., 2013).

Paired sample *t*‐tests were used to test the between‐habitat (impoundments and free‐flowing segments) differences in the mean taxonomic and functional similarities of fish assemblages. The Mantel permutation test was used to test the relationships between changes in taxonomic and functional similarities (taxonomic ΔCS and functional ΔCS, respectively) for abundance‐based data. The significance of each Pearson correlation was calculated using the Mantel test with 9999 permutations (Nekola & White, 1999). Differences in the observed percentages of each quadrant (homogenization or differentiation) were tested using the chi‐square test with the null hypothesis that the proportions were the same. The above analyses were performed using the “*vegan*” package of R software (R Core Team, 2018) (Oksanen et al., 2013).

## 3 RESULTS

**FIGURE 3:**
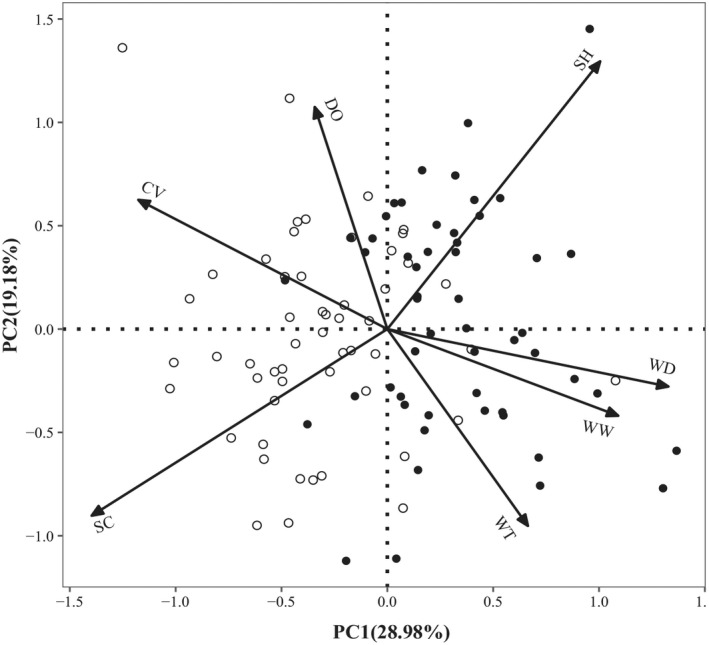
Ordination plot of the principal component analysis (PCA) of sampling sites based on habitat conditions between impoundments and free‐flowing segments. Black and hollow circles represent impoundments and free‐flowing segments, respectively. CV, current velocity; DO, dissolved oxygen; SC, substrate coarseness; SH, substrate heterogeneity; WD, water depth; WT, water temperature; WW, wetted width.

The first and second axes of the PCA explained 28.98% and 19.18% of the habitat's variance, respectively. The impoundments had higher water width, water depth, and lower current velocity and substrate coarseness than the free‐flowing segments (Figure 3). PERMANOVA showed that habitat conditions significantly differed between impoundments and free‐flowing segments (*R* ^2^ = 0.13, *p* < 0.001). However, PERMDISP showed that habitat heterogeneity (average distance to median) did not significantly differ between the two habitat types (ANOVA‐like permutation *F* = 0.89, *p* > 0.05), and that of the impoundments (2.31 ± 1.09 [mean ± standard deviation (SD)]) was marginally higher than that of the free‐flowing segments (2.11 ± 1.08 [mean ± SD]).

### 3.1 Species compositions

Thirty‐one species representing 11 families and 5 orders were collected. Twenty‐nine species occurred in both impoundments and free‐flowing segments, of which the common species were *Zacco platypus*, *Acrossocheilus fasciatus*, and *Ctenogobius sp*. (*FO* > 40%), which were also abundantly dominant (*RA* > 10%), except for *A. fasciatus*. The average species abundance per sample was 46.11 ± 29.43 (mean ± SD) individuals in impoundments and 62.28 ± 39.49 (mean ± SD) individuals in free‐flowing segments, respectively. In addition, *Ctenopharyngodon idella* and *Micropercops swinhonis* (native‐invasive species) were only collected in impoundments, whereas *Leptobotia guilinensis* and *Parasinilabeo assimilis* (native species) only occurred in free‐flowing segments (Table 1).

**TABLE 1:** Species composition, frequency of occurrence (*FO*), and relative abundance (*RA*) of fishes collected in impoundments (*Im*) and free‐flowing segments (*Fr*).

Order/family/species	*FO* (%)		*RA* (%)	
	*Im*	*Fr*	*Im*	*Fr*
**Cypriniformes**				
Cobitidae				
*Cobitis sinensis*	13.21	3.77	4.50	0.09
*Cobitis rarus*	37.74	33.96	5.85	3.94
*Misgurnus anguillicaudatus* ^a^	50.94	37.74	3.76	1.79
*Leptobotia guilinensis*		7.55		0.18
Homalopteridae				
*Vanmanenia stenosoma*	35.85	75.47	2.00	10.06
Cyprinidae				
*Zacco platypus*	94.34	86.79	33.14	37.23
*Acrossocheilus fasciatus*	67.92	62.26	10.35	7.97
*Opasrrichthys bidens*	11.32	11.32	0.41	0.30
*Belligobio nummifer*	13.21	3.77	0.65	0.12
*Rhodeus ocellatus* ^a^	32.08	32.08	5.73	8.00
*Acheilognathus chankaensis*	5.66	5.66	1.27	1.39
*Rhynchocypris oxycephalus*	9.43	13.21	4.91	4.21
*Carassius auratus* ^a^	16.98	13.21	2.62	0.64
*Abbottina rivulars* ^a^	11.32	5.66	1.55	0.42
*Aphyocypris chinensis*	9.43	5.66	0.41	0.85
*Sarcocheilichthys Parvus* ^a^	5.66	5.66	0.53	0.61
*Squalidus argentatus* ^a^	5.66	9.43	0.20	0.55
*Pseudorasbora parva* ^a^	20.75	9.43	1.31	1.06
*Acheilognathus taenianalis* ^a^	1.89	1.89	0.04	0.06
*Onychostoma barbatula*	1.89	7.55	0.08	0.18
*Ctenopharyngodon idella* ^a^	1.89		0.04	
*Parasinilabeo assimilis*		3.77		0.09
**Siluriformes**				
Amblycipitidae				
*Liobagrus styani*	7.55	20.75	0.33	1.24
Bagridae				
*Pseudobagrus truncates*	11.32	16.98	0.86	0.45
**Beloniformes**				
Adrianichthyidae				
*Oryzias sinensis* ^a^	3.77	1.89	0.37	0.06
**Synbranchiformes**				
Synbranchidae				
*Monopterus albus* ^a^	13.21	5.66	0.53	0.09
Mastacembelidae				
*Sinobdella sinensis* ^a^	9.43	11.32	0.33	0.27
**Perciformes**				
Odontobutidae				
*Odontobutis potamophila* ^a^	30.19	28.30	2.25	1.51
*Micropercops swinhonis* ^a^	3.77		0.08	
Gobiidae				
*Ctenogobius sp*.	66.04	79.25	15.83	16.54
Percichthyidae				
*Siniperca chuatsi* ^a^	1.89	3.77	0.08	0.09

^a^ Indicates native‐invasive fish species based on Scott and Helfman (2001) and Liu et al. (2019).

ANOSIM showed that fish assemblage composition strongly overlapped but significantly differed between the impoundments and free‐flowing segments (Global *R* = .04, *p* = .014). Results of the SIMPER analyses showed that the differences in assemblage composition resulted from the abundance changes of nine species between the two habitat types. Compared with the free‐flowing segments, the abundance of four natives (*Z. platypus*, *Ctenogobius sp*., *A. fasciatus*, and *Vanmanenia stenosoma*) and one native‐invasive species (*Rhodeus ocellatus*) decreased in the impoundments, whereas that of three native‐invasive (*Misgurnus anguillicaudatus*, *Odontobutis potamophila*, and *Carassius auratus*) and one native (*Cobitis rarus*) species increased (Table 2).

**TABLE 2:** Contribution of key species to the differences in fish assemblages between the impoundments (*Im*) and free‐flowing segments (*Fr*) based on SIMPER.

Species	*AA*		*AD* (%)	*Con* (%)
	*Im*	*Fr*		
*Zacco platypus*	15.28	23.19	7.75	12.56
*Ctenogobius sp*.	7.30	10.30	7.39	11.97
*Acrossocheilus fasciatus*	4.77	4.96	6.16	9.98
*Vanmanenia stenosoma*	0.92	6.26	6.02	9.75
*Rhodeus ocellatus*	2.64	4.98	4.71	7.62
*Cobitis rarus*	2.70	2.45	4.58	7.41
*Misgurnus anguillicaudatus* ^a^	1.74	1.11	3.62	5.86
*Odontobutis potamophila* ^a^	1.04	0.94	2.93	4.75
*Carassius auratus* ^a^	1.21	0.40	1.90	3.08

*Note*: The cumulative contributions of the species listed were more than 70%.

Abbreviations: AA, average abundance; AD, average dissimilarity; con, contribution.

^a^ Native‐invasive fish species.

### 3.2 Community similarity

**FIGURE 4:**
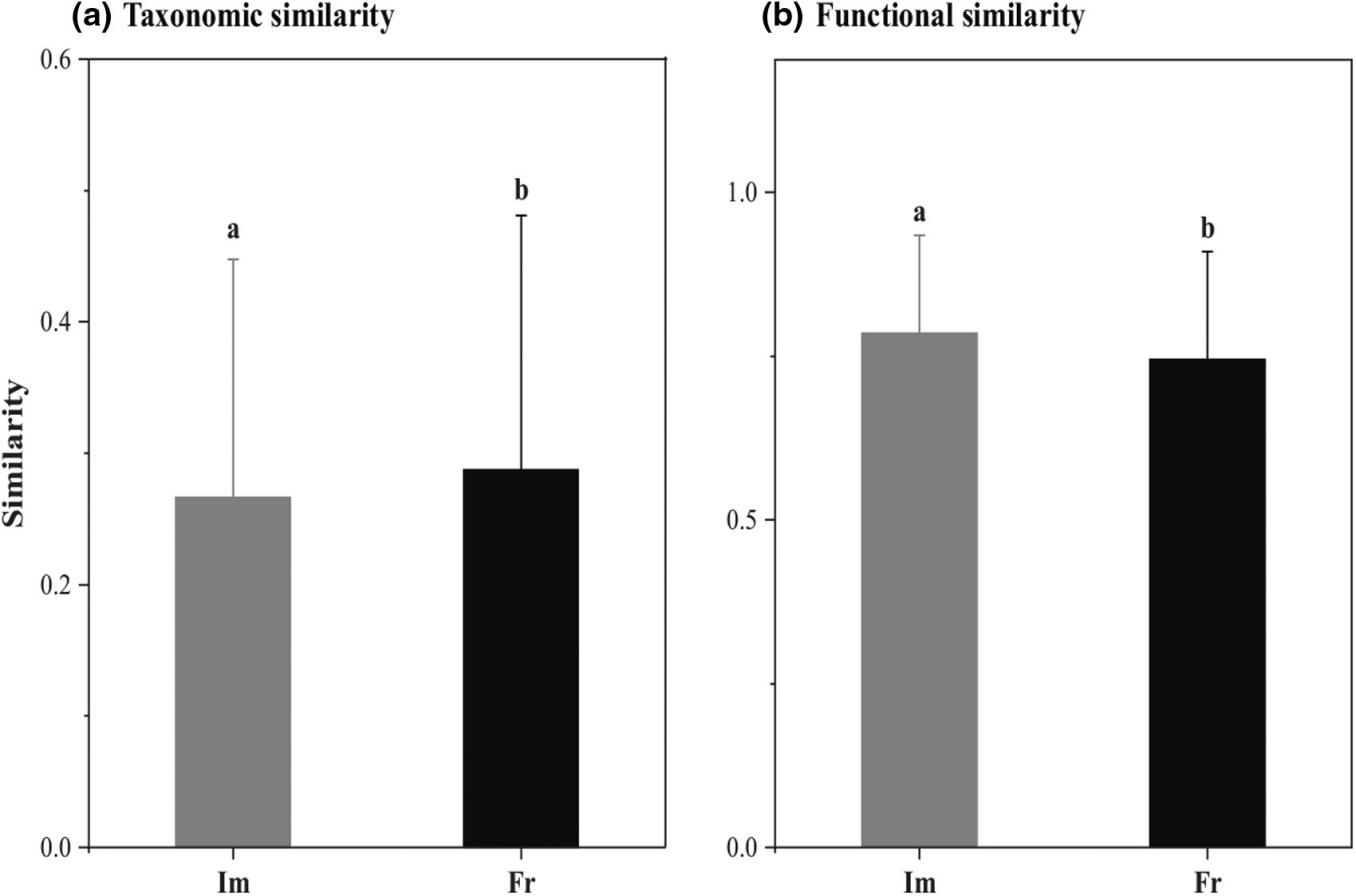
Bar plots showing the mean ± standard deviation for (a) taxonomic similarity, and (b) functional similarity of fish assemblages in the impoundments (*Im*: Gray) and free‐flowing segments (*Fr*: Black). Different letters represent significant differences (*p* < .05) based on paired‐sample *t*‐tests.

Overall, both the taxonomic and functional similarities of fish assemblages differed significantly between impoundments and free‐flowing segments (*p* < .001). Specifically, the taxonomic similarity of fish assemblages was significantly lower in impoundments than that in free‐flowing segments, whereas the functional similarity of fish assemblages was generally higher in impoundments (Figure 4). An opposite trend was detected between the changes in taxonomic and functional similarities, that is, taxonomic differentiation (taxonomic ΔCS: −2.09% ± 21.89%) was accompanied by functional homogenization (functional ΔCS: 4.05% ± 13.03%) in stream fish assemblages.

**FIGURE 5:**
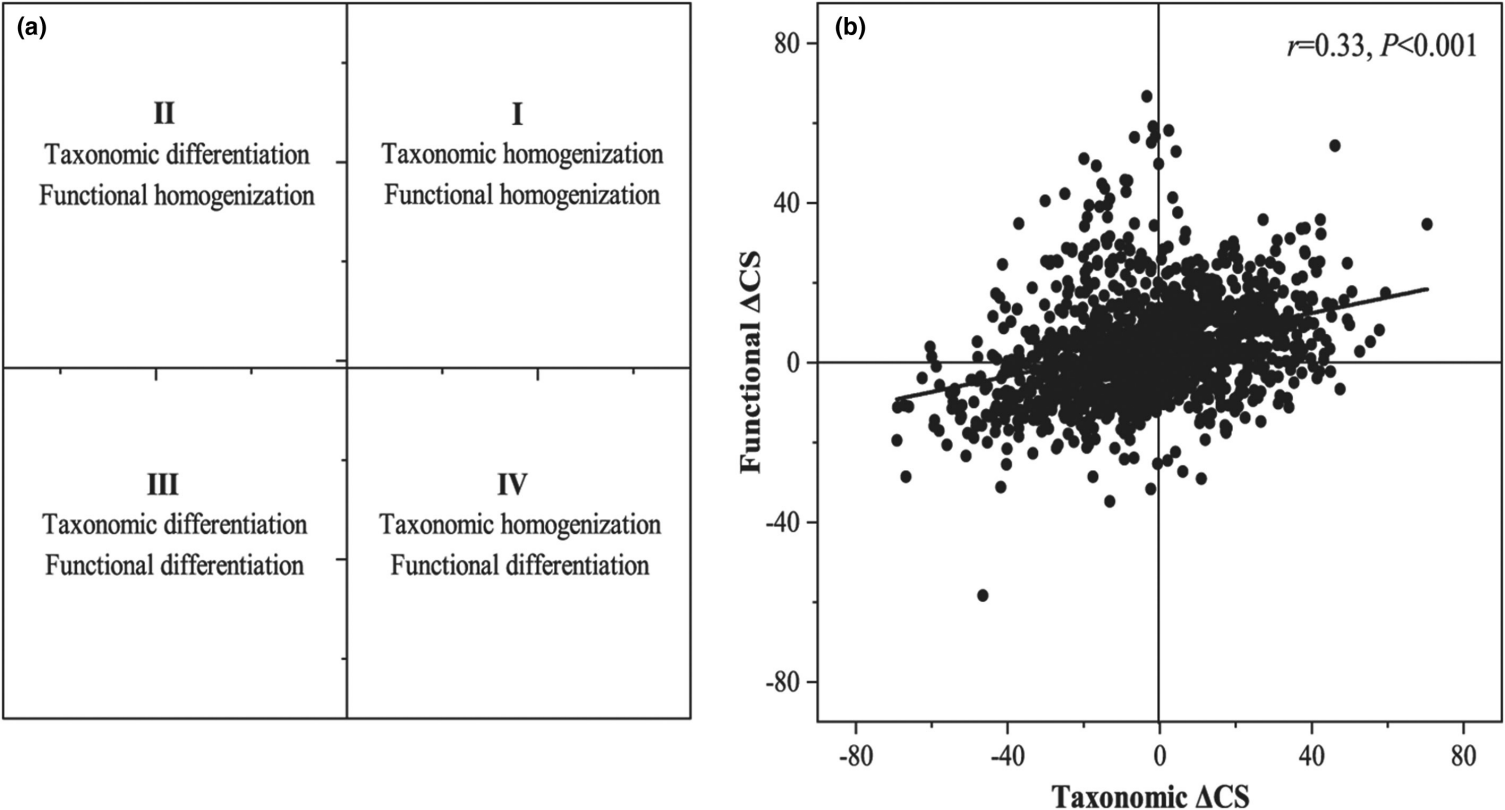
Correlations between changes in taxonomic and functional similarities of fish assemblages (b). Black circles indicate the results from 1378 pairwise assemblages. Pearson correlation (*r*) and significance (*p* value) of the Mantel tests are provided in each quadrant. Each quadrant represents a different combination of taxonomic and functional changes of fish assemblages (a): (I) first quadrant, (II) second quadrant, (III) third quadrant, and (IV) fourth quadrant.

Mantel tests showed that changes in taxonomic and functional similarities were positively correlated (*r* = .33, *p* < .001; Figure 5b). Furthermore, chi‐square tests showed that the observed percentages of taxonomic ΔCS and functional ΔCS (differentiation and homogenization) for pairwise assemblages were statistically significant (*χ* ^2^ = 140.1, *df* = 1, *p* < .001). Among the 1378 pairwise assemblages, 64.73% (892 comparisons) showed uniform changes between taxonomic ΔCS and functional ΔCS, of which 36.14% pairs (quadrant I: 498 comparisons) presented both taxonomic and functional homogenization and 25.11% pairs (quadrant III: 394 comparisons) showed differentiation (Figure 5b and Table 3). However, 35.27% (486 comparisons) showed differences between the taxonomic ΔCS and functional ΔCS. Specifically, 25.11% of pairs (quadrant II: 346 comparisons) showed taxonomic differentiation but functional homogenization, and 10.16% of pairs (quadrant IV: 140 comparisons) presented opposite changes (Figure 5b and Table 3).

**TABLE 3:** Summary of percentages of (Δ) taxonomic and (Δ) functional similarities (i.e., differentiation and homogenization) for pairwise assemblages based on abundance approaches.

Change trend	Functional homogenization	Functional differentiation	Total taxonomic change
Taxonomic homogenization	36.14% (*n* = 498)	10.16% (*n* = 140)	46.30% (*n* = 638)
Taxonomic differentiation	25.11% (*n* = 346)	28.59% (*n* = 394)	53.70% (*n* = 740)
Total functional change	61.25% (*n* = 844)	38.75% (*n* = 534)	

*Note*: Values in parentheses are the number of pairwise assemblages.

## 4 DISCUSSION

Disruption of hydrologic connectivity triggered by dams can affect flow regimes, habitat, and physicochemical parameters of river systems (Hitchman et al., 2018; Tiemann et al., 2004; Yan et al., 2013). Previous studies have shown that low‐head dams may modify local habitats in streams, including deeper water, slower flow, smaller substrate in impoundments upstream, and cause faster flow and larger substrate in plunging areas downstream (Fencl et al., 2015; Li et al., 2021; Yan et al., 2013). Furthermore, PERMDISP analyses suggested that the heterogeneity of local habitat did not significantly differ between the impoundments and free‐flowing segments, and the habitat differentiation in impoundments was marginally higher than that of free‐flowing segments. Habitat conditions are highly heterogeneous along the upstream–downstream gradients of streams, and larger spatial scales encompass greater environmental heterogeneity (López‐Delgado et al., 2019; Vannote et al., 1980). When all spatial units share the same range of habitat characteristics at relatively large scales, common changes in habitat conditions in impoundments (increasing water depth, decreasing flow rate, and decreasing substrate size) may reduce spatial heterogeneity and further cause habitat homogenization among impoundments (Bu et al., 2017). In contrast, dams would result in greater environmental differences and induce habitat differentiation across impoundments at smaller scales (Liu et al., 2019; Smith & Mather, 2013), as the degree to which local habitat is modified often varies with dam size and operation (Li et al., 2021; Poff & Hart, 2002). In this study, all 53 low‐head dams surveyed were located within first‐order headwater streams in the Wannan Mountains; therefore, marginal habitat differentiation in impoundments was observed at finer spatial scales.

In terms of species composition, among 31 species, 29 occurred in both the impoundments and free‐flowing segments, and only a few species were gained and lost. Earlier studies have shown that the transformation of lotic to lentic habitats in impoundments created by low‐head dams can not only cause the population of native fish species to decline and even locally extinguish but also favor the occurrence and spread of native invaders into upland streams (Chu et al., 2015; Liu et al., 2019; Smith et al., 2017). Although a fraction of native species is lost and native‐invasive species are gained in impoundments, a large number of the remaining species (especially abundant species) often tend to decline or increase in abundance (Liu et al., 2019; Tiemann et al., 2004). In this study, we also found significant changes in community composition between the impoundments and free‐flowing segments. This was possible because changes in species abundance can affect the degree to which communities differ in composition, despite having pairwise communities with the same species identities (Barwell et al., 2015; Cassey et al., 2008).

Our results demonstrated the overall tendency of fish taxonomic differentiation for abundance‐based approaches in impoundments relative to free‐flowing segments (Figure 4a), which is consistent with the marginal habitat differentiation observed in impoundments. Surprisingly, taxonomic homogenization is often observed at relatively large scales (inter‐ecoregion or basins), which is the result of both the establishment of widespread species and the loss of native species (Daga et al., 2020; Kirk et al., 2020; Toussaint et al., 2016). However, our results are inconsistent with those of previous studies conducted at broader spatial scales. The first signs of changes in fish diversity patterns affected by low‐head dams may be mainly reflected in abundance changes (Fencl et al., 2017; Liu et al., 2019; Tiemann et al., 2004). If the number of shared species between communities does not change, but the abundance does, the Bray–Curtis similarity index based on abundance approaches will show changes that depend on the magnitude of shifts in abundance (Baselga, 2013; Cassey et al., 2008). In addition to the decline in abundance of shared dominant species, the dominant species became rarer (*V. stenosoma*) and the abundance of some unique species markedly increased (*M. anguillicaudatus* and *C. auratus*) in impoundments, which may result in taxonomic differentiation of current fish assemblages based on abundance approaches. Additionally, previous studies have revealed that fish assemblages undergo a transition phase from taxonomic differentiation to homogenization over time (Petesse & Petrere, 2012; Pool & Olden, 2012). Given that the expansion and colonization of widespread species may require considerable time to negatively affect native species (Ding et al., 2017), the observed taxonomic differentiation of current fish assemblages (a short‐term phenomenon) is probably an early warning sign of further homogenization.

We found that changes in the functional similarity of fish assemblages tended to demonstrate homogenization (Figure 4b), which showed opposite trends to changes in taxonomic similarity. Such patterns are often the result of a decrease in the abundance of shared native species with unique functional roles, and the establishment and colonization of widespread species with similar functional roles (Olden & Rooney, 2006; Pool & Olden, 2012). Previous research in this region (Li et al., 2021) has shown that some native species (typical lotic species) could bring unique functional traits, and native‐invasive and native species potentially have functional redundancy. In other words, the abundance of native species with unique functional traits decreased sharply, and some native‐invasive species increased, both of which contributed to functional homogenization despite diverging taxonomies (Pool & Olden, 2012). More importantly, even if a significantly positive correlation was observed between changes in mean taxonomic and functional similarities (Figure 5b), approximately one‐third of the pairwise assemblages presented contrasting outcomes (Table 3). The pattern of fish faunal homogenization and differentiation largely depends on spatiotemporal changes in species abundance, and ecological functions of these species are gained and lost among communities (Cassey et al., 2008; Socolar et al., 2016; Villéger et al., 2014). Taxonomic differentiation but functional homogenization can occur when shared native species with unique functional traits decline in range or abundance, whereas taxonomic homogenization but functional differentiation can occur when unshared species with similar traits are lost (Campbell & Mandrak, 2020; Villéger et al., 2014). This discrepancy between changes in taxonomic and functional similarities could be explained by functional redundancy among different species, which is supported by previous studies (Su et al., 2015; Villéger et al., 2014).

## 5 CONCLUSIONS

Our study reveals the patterns and extent of fish homogenization and differentiation that are induced by low‐head dams in subtropical streams in China. In this study, we found that taxonomic differentiation accompanied functional homogenization in stream fish assemblages because of the decrease in the abundance of shared native species with unique functional traits. More importantly, these results show the importance of population abundance in structuring fish faunal homogenization and differentiation at small scales, especially when the major differences among assemblages are in species abundance ranks rather than species identities. Additionally, our study showed that changes in taxonomic similarity cannot be used to predict changes in functional similarity. Furthermore, considering the dynamic processes of biotic homogenization and differentiation, the taxonomic differentiation of current fish assemblages observed in this study is probably an early warning sign of further homogenization in regions where native species are completely predominated. Therefore, future studies should continue monitoring to protect existing biodiversity and mitigate the ecological effects of low‐head dams.

## AUTHOR CONTRIBUTIONS

**Qiang Li:** Conceptualization (equal); investigation (equal); methodology (equal); writing – original draft (lead). **Yuzhou Zhang:** Conceptualization (equal); investigation (equal); methodology (equal). **Ruolan Wang:** Formal analysis (equal); methodology (equal); software (equal). **Ling Chu:** Conceptualization (equal); investigation (equal); methodology (equal). **Yuru Li:** Investigation (equal). **Yunzhi Yan:** Conceptualization (equal); data curation (equal); investigation (equal); project administration (supporting); writing – review and editing (lead).

## CONFLICT OF INTEREST

We declare that we have no conflict of interest.

## Supporting information


Appendix S1

Figure S1

## Data Availability

The data used to support the findings of this study are available on the Data Dryad Digital Repository, https://doi.org/10.5061/dryad.1g1jwsv0c.
