# Supplementary material for: Low‐head dams induce biotic homogenization/differentiation of fish assemblages in subtropical streams
Source: Ecol Evol. 2022 Jul 30;12(8):e9156. doi: 10.1002/ece3.9156 (PMC9338443; doi:10.1002/ece3.9156)
Supplement: Supplementary file 1 — Appendix S1 [file ECE3-12-e9156-s001.docx]

**TABLE S1** List of 7 functional traits derived from 11 morphological measures with abbreviations and calculations. See FIGURE S1 for definitions

| Functional Trait | Equation | Ecological meaning | References |
| --- | --- | --- | --- |
| Eye size | $\frac{\text{ED}}{\text{HD}}$ | Prey detection | Boyle & Horn, 2006 |
| Oral gape position | $\frac{\text{M}\text{O}}{\text{HD}}$ | Feeding position in the water column | Sibbing & Nagelkerke, 2001 |
| Gut length | $\frac{\text{GL}}{\text{B}\text{L}}$ | Processing of energy poor resources such as vegetation and detritus | Kramer & Bryant, 1995 |
| Body transversal shape | $\frac{\text{BD}}{\text{BW}}$ | Vertical position in the water column and hydrodynamism | Sibbing & Nagelkerke, 2001 |
| Body depth | $\frac{\text{BD}}{\text{B}\text{L}}$ | Swimming ability in water | Sibbing & Nagelkerke, 2001 |
| Oral gape shape | $\frac{Md}{Mw}$ | Method to capture food items | Karpouzi & Stergiou, 2003 |
| Caudal peduncle throttling | $\frac{CFd}{CPd}$ | Caudal propulsion efficiency through reduction of drag | Webb, 1984 |


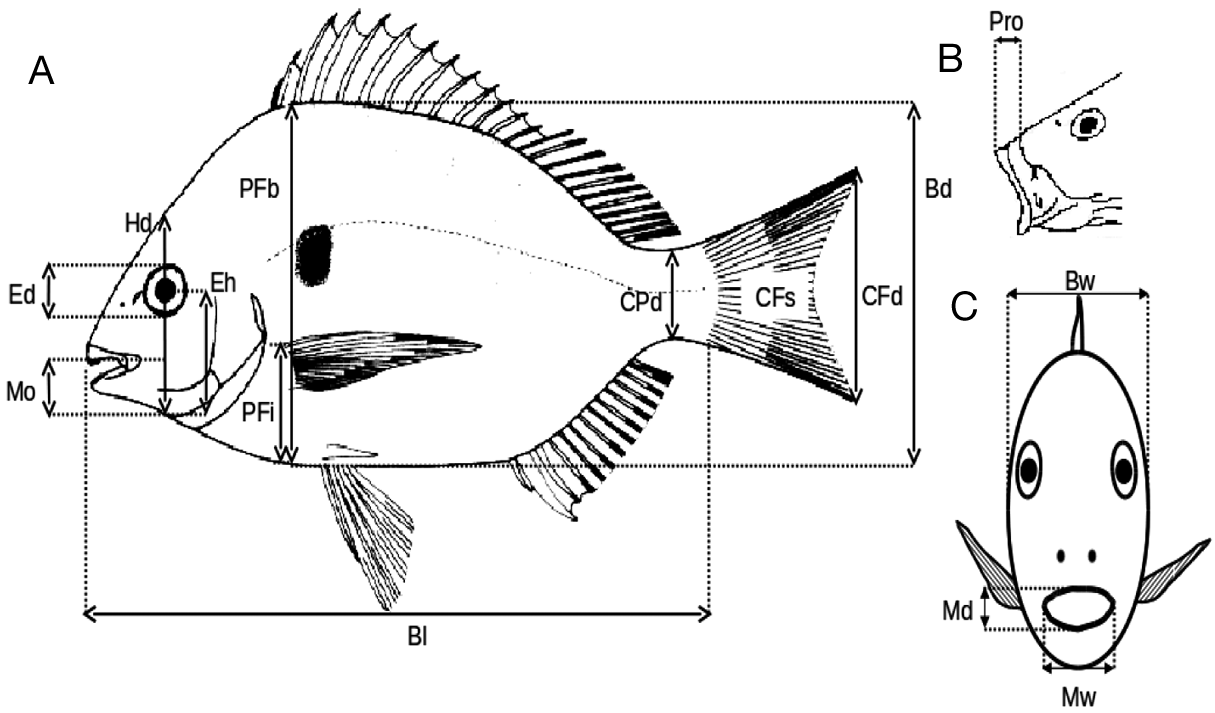


**FIGURE S1.** Illustration of the morphological traits: (A) Side view. (B) Mouth gape. (C) Front view. BD = Body depth; Bl = Body standard; BW = Body width; CFd = caudal fin depth; CPd = caudal peduncle minimal depth; Ed = eye diameter; Eh = distance between the bottom of the head and the eye center along the head depth axis; Hd = head depth along the vertical axis of the eye; Md = mouth depth; Mw = mouth width; PFb = body depth at the level of the pectoral fin insertion; PFi = distance between the insertion of pectoral fin and the bottom of the body; Pro = stretched protrusion length. Also measured, but not shown in diagrams —— GRl: gill raker length; Gl: gut length (adapted from Albouy et al., 2011).

**REFERENCES**

Albouy, C., Guilhaumon, F., Villéger, S., Mouchet, M., Mercier, L., Culioli, J. M., Tomasini, J. A., Le Loch, F., & Mouillot, D., (2011). Predicting trophic guild and diet overlap from functional traits: Statistics, opportunities and limitations for marine ecology. *Marine Ecology Progress Series*, 436, 17-28. https://doi.org/10.3354/meps09240

Boyle, K. S., & Horn, M. H. (2006). Comparison of feeding guild structure and ecomorphology of intertidal fish assemblages from central California and central Chile. *Marine Ecology Progress Series*, 319, 65-84. https://doi.org/10.3354/meps319065

Karpouzi, V., & Stergiou, K. (2003). The relationships between mouth size and shape and body length for 18 species of marine fishes and their trophic implications.*Journal of Fish Biology*, 62, 1353-1365. https://doi.org/10.1046/j.1095-8649.2003.00118.x

Kramer, D. L., & Bryant, M. J. (1995). Intestine length in the fishes of a tropical stream. II. Relationships to diet—the long and short of a convoluted issue. *Environmental Biology of Fishes*, 42, 129-141. https://doi.org/10.1007/BF00001991

Sibbing, F. A., & Nagelkerke, L. A. J. (2001). Resource partitioning by Lake Tana barbs predicted from fish morphometrics and prey characteristics. *Reviews in Fish Biology and Fisheries*, 10, 393-437. https://doi.org/10.1023/A:1012270422092

Webb, P. W. (1984). Form and function in fish swimming. *Scientific American*, 251, 72-82. https://doi.org/10.1038/scientificamerican0784-72
