# Supplementary material for: Low‐head dams induce biotic homogenization/differentiation of fish assemblages in subtropical streams
Source: Ecol Evol. 2022 Jul 30;12(8):e9156. doi: 10.1002/ece3.9156 (PMC9338443; doi:10.1002/ece3.9156)
Supplement: Supplementary file 2 — Figure S1 [file ECE3-12-e9156-s002.docx]

**
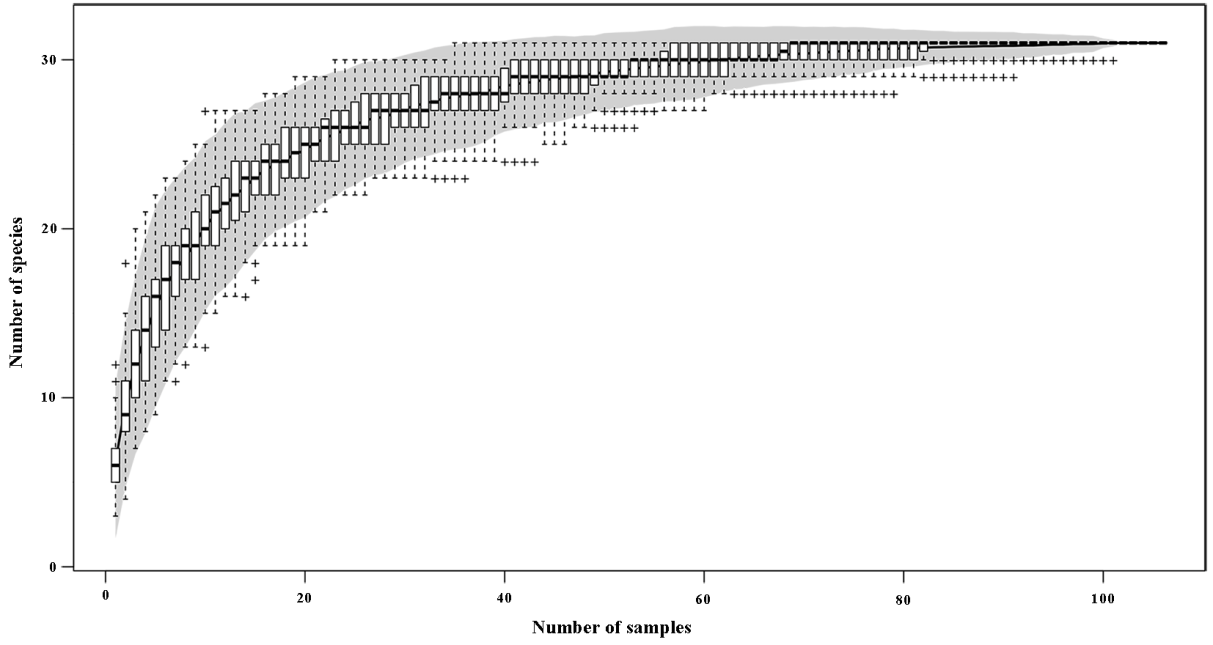
**

**FIGURE S1.** Species accumulation curve for the fishes recorded in the first-order headwater streams of the Wannan Mountains. This result indicates sampling effort was sufficient to collect most of the species.
